# Computational simulation of liver fibrosis dynamics

**DOI:** 10.1038/s41598-022-18123-w

**Published:** 2022-08-18

**Authors:** Misa Yoshizawa, Masahiro Sugimoto, Minoru Tanaka, Yusuyuki Sakai, Masaki Nishikawa

**Affiliations:** 1 Department of Chemical System Engineering, University of Tokyo, Tokyo, Japan; 2 Institute of Medical Science, Tokyo Medical University, 6-1-1, Shinjuku, Tokyo, 160-0022 Japan; 3 Institute for Advanced Biosciences, Keio University, Yamagata, Japan; 4 Department of Regenerative Medicine, Research Institute, National Centre for Global Health and Medicine, Tokyo, Japan

**Keywords:** Systems biology, Biochemical networks, Computer modelling, Dynamical systems, Numerical simulations

## Abstract

Liver fibrosis is a result of homeostasis breakdown caused by repetitive injury. The accumulation of collagens disrupts liver structure and function, which causes serious consequences such as cirrhosis. Various mathematical simulation models have been developed to understand these complex processes. We employed the agent-based modelling (ABM) approach and implemented inflammatory processes in central venous regions. Collagens were individually modelled and visualised depending on their origin: myofibroblast and portal fibroblast. Our simulation showed that the administration of toxic compounds induced accumulation of myofibroblast-derived collagens in central venous regions and portal fibroblast-derived collagens in portal areas. Subsequently, these collagens were bridged between central-central areas and spread all over areas. We confirmed the consistent dynamic behaviour of collagen formulation in our simulation and from histological sections obtained via in vivo experiments. Sensitivity analyses identified dead hepatocytes caused by inflammation and the ratio of residential liver cells functioned as a cornerstone for the initiation and progression of liver fibrosis. The validated mathematical model demonstrated here shows virtual experiments that are complementary to biological experiments, which contribute to understanding a new mechanism of liver fibrosis.

## Introduction

The liver plays a central role in various metabolic processes and contributes to the homeostasis of the body^1^. Although the regenerative capacity of the liver is a crucial characteristic of this organ, several liver disorders and diseases weaken this ability and disrupt liver homeostasis, which often initiates liver fibrosis^2^. Liver fibrosis is defined as an excessive accumulation of connective tissue components deposited by fibroblastic cells, and fibrotic tissue often disrupts the physiological structure and organ function^3,4^. In addition, continuous and repetitive tissue injury induces liver fibrosis and even cirrhosis, an end-stage liver fibrosis. Cirrhosis is generally irreversible and requires organ transplants, and approximately 40,000 deaths per year in the United States are attributed to cirrhosis^5^. The key to decreasing the number of fatalities is preventing the progression of liver fibrosis, which occurs over years and decades. Therefore, early detection and understanding of the mechanisms and dynamics of liver fibrosis progression are crucial.

Tissue injury risk factors and triggers causing fibrosis are organ dependent; however, the core pathways and mechanisms are often similar in different organs^6–9^. Dead cells from tissue injury release damage-associated molecular patterns (DAMPs). DAMP includes various pro-inflammatory endogenous molecules, such as high mobility group box 1 (HMGB1), adenosine triphosphate (ATP), extracellular cold-inducible RNA-binding protein (eCIRP), histones, heat shock proteins (HSPs), extracellular RNAs (exRNAs), and cell-free DNA (cfDNA)^10^. Although the composition of DAMPs may vary depending on the type of cell death, all dead cells somewhat contribute to the release of DAMPs and initiate a variety of inflammatory responses as a reparative process^11,12^. Generally, this inflammation recruits and polarises macrophages, and these polarised macrophages release profibrotic mediators, including transforming growth factor-β (TGF-β) and platelet-derived growth factor (PDGF), and activate signalling pathways. These mediators and other signalling pathways, such as Wnt and hedgehog, play a critical role in activating fibroblasts, which are the primary producers of extracellular matrix (ECM) proteins. Excessive deposition of ECMs induces stiffness and changes in organ structures, causing further damage to the organ via hypoxia. In addition, this self-amplifying loop leads to the progression of fibrosis.

In the liver, residential macrophages (Kupffer cells) and infiltrating monocyte-derived macrophages recruited from circulating blood promote fibrogenesis via secretion of TGF-β and other mediators. These mediators activate hepatic myofibroblasts to secrete the ECM. Although it is difficult to determine the origin of hepatic myofibroblasts in clinical liver disease, hepatic stellate cells (HSCs), portal fibroblasts, and bone marrow-derived cells have been identified as the origin^13–15^. Specifically, activated HSCs and activated portal fibroblasts are the primary producers of ECMs in experimental models of liver fibrosis^16^.

Histologically, liver fibrosis exhibits distinct patterns depending on the aetiology^17^. Two general types of aetiology are hepatotoxic and cholestatic injuries. Hepatotoxic injury is caused by chronic viral infections, drugs, alcohol, and metabolic syndrome. The factors affect the pericentral region of the liver lobules. Subsequently, this activates HSCs and progresses from the pericentral area. Meanwhile, cholestatic injury is caused by bile flow disruption from biliary obstruction or impaired secretion by hepatocytes. Therefore, it activates near portal fibroblasts and progresses from the periportal region^18^. Thus, the composition of myofibroblasts also varies, depending on the aetiology of liver fibrosis^16^. Through liver injury and associated inflammation, liver fibrosis develops central-central, portal-portal, and mixed bridging depending on the aetiology and severity, and progresses to a decompensated phase, cirrhosis, which leads to death or the need for liver transplantation^19^.

Despite intensive studies, the mechanisms and dynamics of liver fibrosis are not fully elucidated. The progression of liver fibrosis involves complex interactions of cells and various factors. It occurs for decades in humans and months and years in animals. Therefore, an accurate diagnosis of liver fibrosis remains challenging^20–22^. As research tools, liver organoids and other in vitro cell culture models have made significant progress recently^23–25^. However, they are unable to reproduce the required complexity and dynamics of the disease^26,27^. In animals, several hepatic fibrosis models have been developed and have been indispensable tools to enhance our understanding^24,25,28,29^. However, there is no perfect model that can replicate the process of human liver fibrosis. Additionally, the results of animal experiments require careful interpretation for correct translation for humans because of their genetic and mechanistic differences^25^. As a complemental approach, computer-based research and simulation experiments have recently shown promising results. Compared with in vivo and in vitro methods, they have substantial advantages in generating and validating hypotheses. For liver fibrosis, to recapitulate overall cellular population dynamics based on local interactions, agent-based models (ABMs) have been used^30–34^. Dutta-Moscato et al. developed an ABM model that simulates the progression of drug-induced liver fibrosis, represented by tetrachloromethane (CCl_4_) exposure^31^. This study improved their model to reproduce fibrosis progression more accurately. The histological patterns were compared with experimental data from CCl_4_-treated rats, and two theoretical anti-fibrotic therapies were investigated in silico. We distinguished portal fibroblasts and HSC-derived myofibroblasts based on in vivo observations and succeeded in reproducing the dynamic transition of fibrosis.

## Methods

### Model design

Here, we modified Dutta-Moscato’s model^31^. The diagram depicting the agent-based model and examples of simulated reactions and transitions among agents are shown in Fig. S1. Briefly, the developed mathematical model simulated the dynamisms of liver components within two-dimensional (2D) liver lobules. The space was composed of grids, and cell agents and components were located on each grid.

The liver components were implemented as various agents. Cell agents were:

1. Two states of parenchymal cells, hepatocytes, and dead cells.
2. Kupffer cells (KCs) as residential macrophages.
3. HSC, myofibroblasts, and portal fibroblasts as collagen-producing cells.

The model also included portal and septa agents.

Hepatocytes, dead cells, portal, septa, and collagen are agents that physically occupy an allocated space and exclude each other from their space. KC, HSC, myofibroblasts, and portal fibroblasts are agents that do not occupy a space and can thus exist in the same space with another agent (Fig. S1b). KC, HSC, and myofibroblasts are distributed randomly in the lobule space (Fig. S2). KCs are produced by the portal area and make the undirected random movement to neighbouring grids (Fig. S1b).

All these agents identify other agents when they collide and react based on predetermined rules, e.g. KCs phagocytise dead cells and free the space. Collision is defined as the event when different agents occupy the same grid in the 2D liver lobules (Fig. S1b).

Some agents secrete several diffusion components, e.g., HMGB1, tumour necrosis factor-α (TNF-α), and TGF-β. To simulate this diffusion, we designed a simple discrete approximation in which each data layer grid shares a given percentage of its value with its eight neighbour grids in the 2D liver lobules^35^. These diffusion components affect other agents in both layers and decrease with time^36^.

All agents have an age property and become extinct when the age reaches pre-defined limits. A hepatocyte cell is transformed to dead cells when the concentration of CCl_4_ or TNF-α in the grid where the hepatocyte cell exists is higher than the threshold value (Fig. S1a). KCs phagocytose dead cells when these cells exist on the same grid, and the dead cell disappears, with blank space being assigned to that grid location (Fig. S1a). Hepatocytes monitor the surrounding spaces and replicate themselves to fill in a vacant space (Fig. S1b). However, they cannot be replicated multiple times in collagen-rich environments.

KCs show the subtype transition from M1 to M2, which secrete TNF-α and TGF-β, respectively^37^. In this model, activated KCs were programmed to first secrete TNF-α and TGF-β thereafter.

### Modification of the mathematical model

The modification of the mathematical model is described here (Fig. S2). Based on in vitro mechanisms^38^, we implemented two types of collagens produced by myofibroblasts and portal fibroblasts. HSCs and myofibroblasts were set to not migrate from their initial position, whereas in Dutta-Moscato’s model, collagens were implemented as a single agent and HSC and myofibroblasts moved randomly and produced collagens when they reached the periportal area.

The algorithm to produce HMGB1 was also modified. HMBG1 is secreted from necrotic cells and to a much lesser degree, from apoptotic cells^10^. Therefore, we implemented HMBG1 secretion from toxic compound-induced cell death, i.e., necrosis and not those from inflammation (TNF-α)-induced cell death, i.e., apoptosis (Fig. S4), while dead cells secreted HMGB1 in Dutta-Moscato’s model^31^.

**Figure 1:**
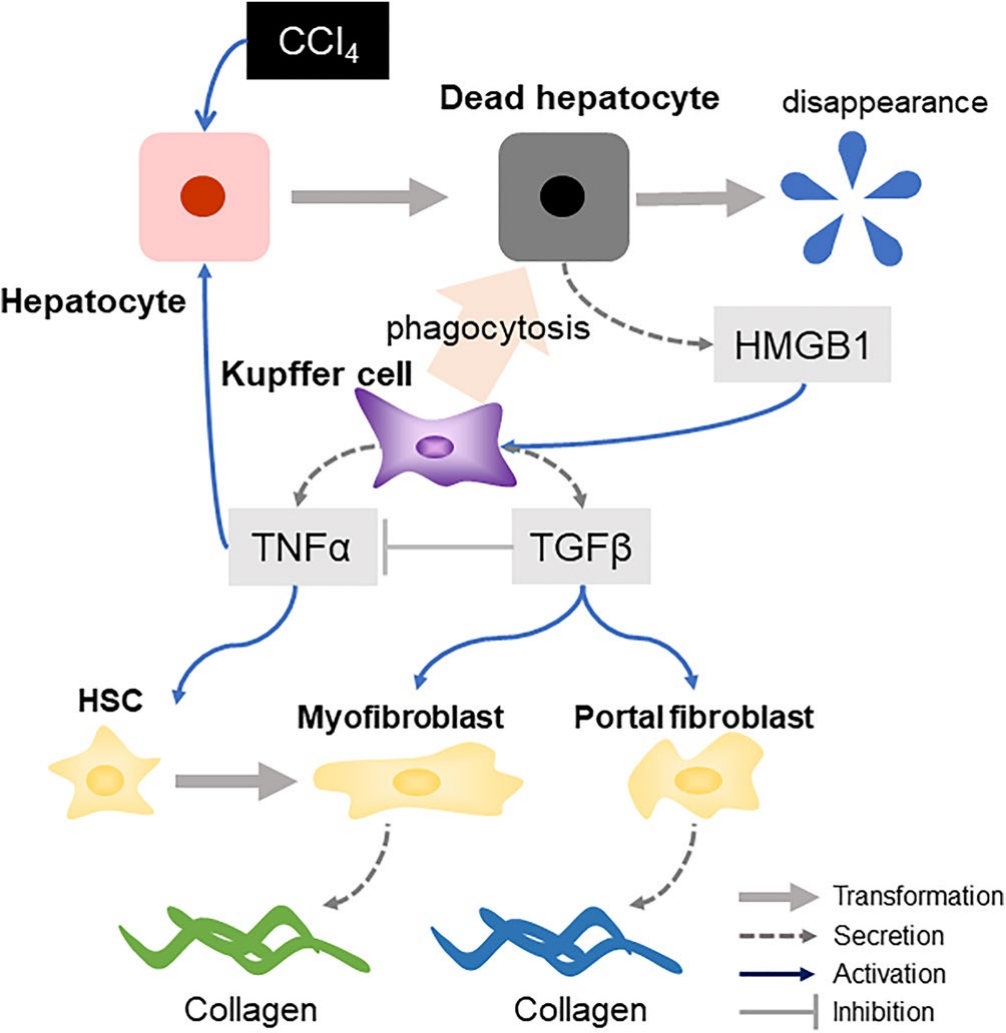
Concept of the developed model. (1) Hepatocytes exposed to toxic compounds (CCl_4_) become dead cells. (2) Kupffer cells are activated by the phagocytosis of dead cells and secrete TNF-α and TGF-β. Insufficient clearance of dead cells causes accumulation of HMGB1, which also activates Kupffer cells. (3) HSCs are transformed into myofibroblasts when the concentration of TNF-α exceeds the pre-defined threshold. (4) Myofibroblasts and portal fibroblasts secrete collagens when the concentration of TGF-β exceeds the pre-defined threshold. Here, two types of collagens were implemented separately depending on their origin.

The summary of the process of liver fibrosis implemented in this study is depicted in Fig. 1. This model focused on liver fibrosis caused by toxic compounds which undergo metabolic activation.

1. Hepatocytes exposed to toxic compounds (CCl_4_) are transformed into dead cells primarily in the central vein area.
2. KCs are activated by phagocytosis of dead cells or by HMGB1 released from dead cells and secrete TNF-α and TGF-β.
3. HSCs are transformed into myofibroblasts by TNF-α.
4. Myofibroblasts and portal fibroblasts produce collagens upon TGF-β exposure.

KCs were randomly located at the initial condition of the simulation as residential macrophages. Macrophages recruited from circulating blood were also implemented as KC agents as they exhibit the same functions in this simulation.

The simulation model was developed on SPARK (ver. 1.2.003, https://github.com/monadius/spark-abm/releases/tag/1.2.003). This library works on Java 8 updates 321 (64-bit) (https://www.java.com/en/download/manual.jsp).

The numerical experiments were conducted on Windows 10 Home OS and Intel(R) Core(TM) i7-1165G7 @ 2.80 GHz 2.80 GHz CPU. The source code and instructions were available at https://www.mip.tokyo-med.ac.jp/research.

### Ethics statement

The study was conducted with the approval of the National Center for Global Health and Medicine (no. 20143-Apr/5/2021). All animal experiments adhered to the ARRIVE Guidelines and were carried out with the Institutional Regulation for Animal Experiments at the National Center for Global Health and Medicine.

### Induction of liver fibrosis in mice

C57BL/6 mice were obtained from CLEA Japan (Tokyo, Japan) and maintained on a 12-h light/dark cycle with free access to a standard chow diet. Liver fibrosis was induced by repeated intraperitoneal injection of CCl_4_ (Wako Pure Chemical, Osaka, Japan), twice a week for 4 weeks. CCl_4_ was diluted to 20% in corn oil (Wako) and injected into mice at a dosage of 1 mL/kg. Livers were harvested 3 d after the final CCl_4_ injection.

## Results

### Dynamics of mathematical simulations

**Figure 2:**
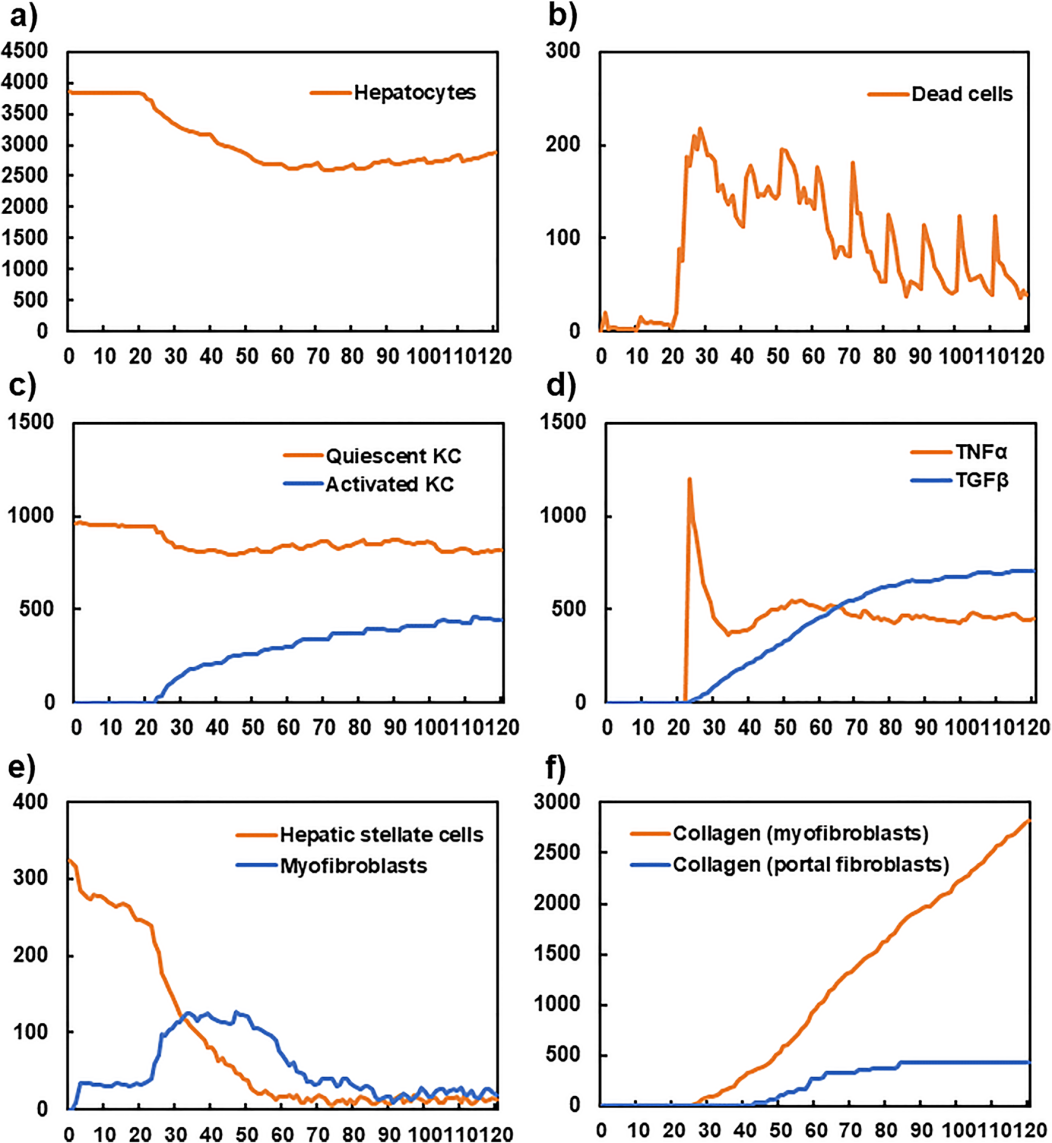
Simulated time-courses of components of the developed model. The X-axis indicates the time step. Y-axes show the number of cells and molecules. Hepatocytes (**a**), dead cells (**b**), quiescent and activated Kupffer cells (**c**), TNF-α and TGF-β (**d**), HSCs and myofibroblasts (**e**), and myofibroblast- and portal fibroblast-derived collagens (**f**).

During initialisation of the simulation, the lobule spaces with filled hepatocytes were structured, and KC, HSC, and myofibroblasts were randomly placed in the spaces (Fig. S2). The movements and reactions of each agent with diffusion of components were calculated with each step (Fig. S1). Figure 2 shows the time-course up to 120 steps of each component in the developed mathematical model. Toxic compounds were administered every ten steps up to 120 steps. Figure 2a shows the time-trajectory of the number of hepatocytes. The number decreased after 20 steps and remained at almost the same level after 60 steps, indicating the balance between cell death due to the toxicity and proliferation of the remaining cells. Figure 2b shows the time-trajectory of dead cells. There was a rapid increase between steps 20 and 30, with a gradual decrease thereafter. It peaked at every ten steps, synchronising with the injection of toxic compounds.

Figure 2c shows the relationship of the number of quiescent and activated Kupffer cells. After approximately 25 steps, activated Kupffer cells emerged and the number of both types of Kupffer cells became relatively stable after approximately 50 steps. This trend was consistent with that of dead cells, as Kupffer cells are activated by phagocytosis of dead cells.

Figure 2d depicts the time-courses of TNF-α and TGF-β. The concentration of TNF-α exhibited a sharp peak at approximately step 25 and became stable thereafter. The concentration of TGF-β increased from step 25 and became stable after step 70. The rapid increase of TNF-α represented the initial activation of Kupffer cells in Fig. 2c, and the trend of TNF-α and TGF-β represented the phenotypic transition of activated Kupffer cells from the M1 to M2 subtype, which secrete TNF-α and TGF-β, respectively.

Figure 2e shows the time-course of HSCs and myofibroblasts. HSCs gradually decreased until step 25, then exhibited a rapid decrease between steps 25 to 50, and stabilised after step 50. Myofibroblasts increased from step 0 to 5, then exhibited a rapid increase from step 25 to 30, and gradually decreased after step 50, stabilising after step 90. This decrease after step 50 was attributed to the characteristics of myofibroblasts which die upon collagen production. Overall, rapid transitions of HSCs and myofibroblasts after step 25 were consistent with the sharp increase of TNF-α at step 25 (Fig. 2e), as HSCs are transformed to myofibroblasts by TNF-α.

Figure 2f shows the time-course of collagens produced by myofibroblasts and portal fibroblasts. The collagens produced by myofibroblasts increased from step 25, whereas the collagens produced by portal fibroblasts increased from step 40 and became stable after 60 steps. Myofibroblasts and portal fibroblasts were compelled to produce collagens when the concentration of TGF-β became higher than the pre-defined threshold. Myofibroblasts-derived collagens increased with the concentration of TGF-β (Fig. 2d).

### Evaluation of the validity of the developed mathematical model

**Figure 3:**
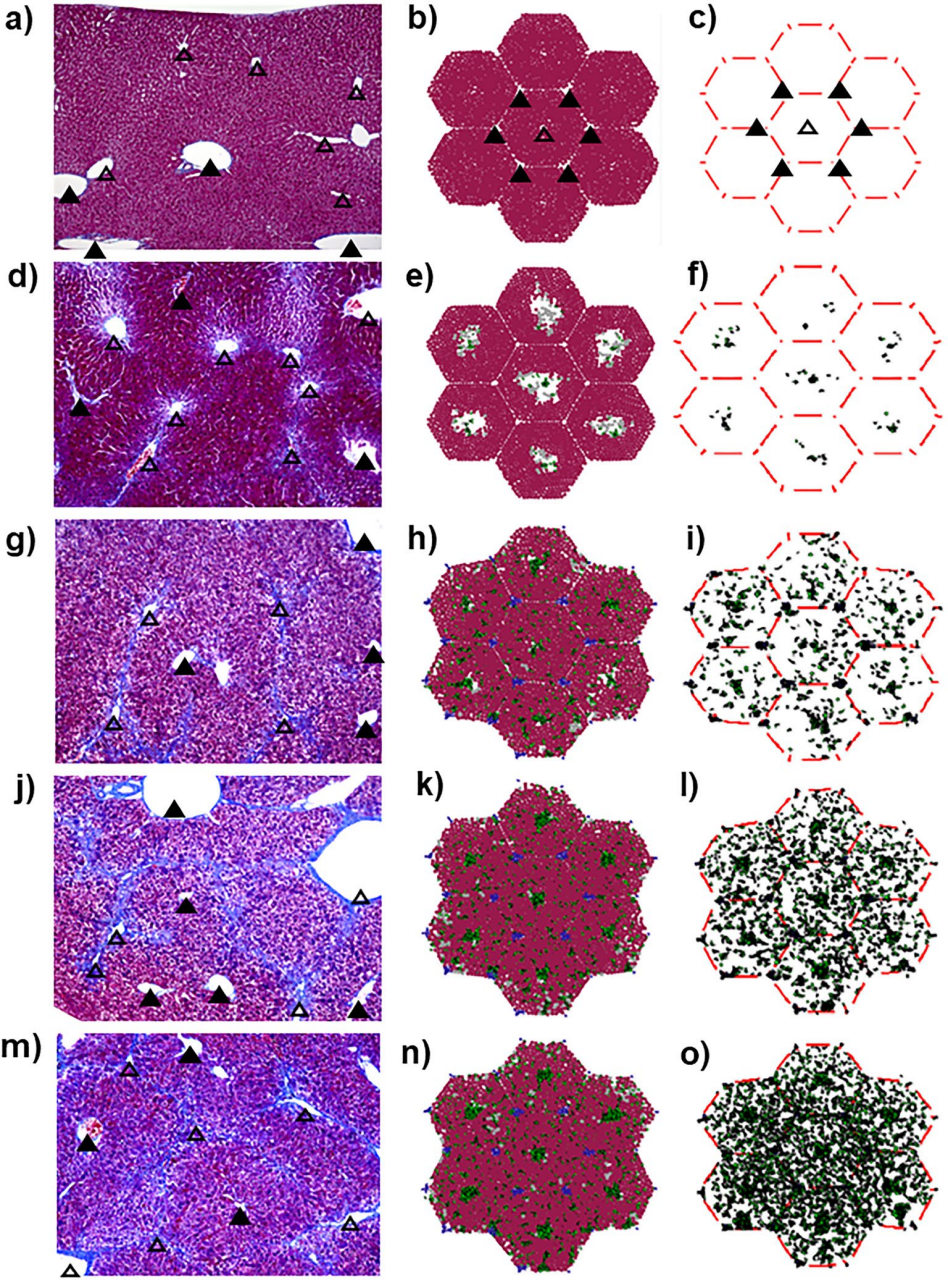
The time-dependent observations of liver lobules. Left panels: experimentally obtained 2-dimensional images of tissue sections (**a**, **d**, **g**, **j**, and **m**). Centre panels are computationally simulated results (**b**, **e**, **h**, **k**, and **n**). The right panels also show simulated results without hepatocytes (**c**, **f**, **i**, **l**, and **o**). The filled and open triangles indicate portal and central veins, respectively. In the simulation results, each agent is shown in different colours; hepatocytes (brown), dead cells (grey), and collagens secreted from myofibroblasts and portal fibroblasts (blue and green).

The simulated results were compared with the in vivo tissue sections to validate the developed model. The 2-D tissue section (Fig. 3, left column) and simulated images (Fig. 3, centre and right columns) are shown. The collagen formulation at five different time points of fibrosis development was compared.

Experimental results shown in the left column started from a healthy liver section without fibrosis (Fig. 3a). Firstly, collagens were produced at the central vein area (Fig. 3d). Secondly, collagens were spread out and extended towards portal vein areas (Fig. 3g). Finally, collagens were bridged among centre vein areas (Fig. 3j,m). Simulations also revealed similar collagen formulation dynamics. Starting with the initial healthy stage (Fig. 3b,c), myofibroblasts-derived collagens emerged in the central vein area (Fig. 3e,f). Subsequently, besides further accumulation of myofibroblasts-derived collagens, portal fibroblast-derived collagens also emerged (Fig. 3h,i). Finally, collagens were bridged among centre vein areas (Fig. 3k,l,n,o). These similarities in collagen formulation dynamics confirmed the validity of the developed model.

### Effect of intervals of toxic compound injection

**Figure 4:**
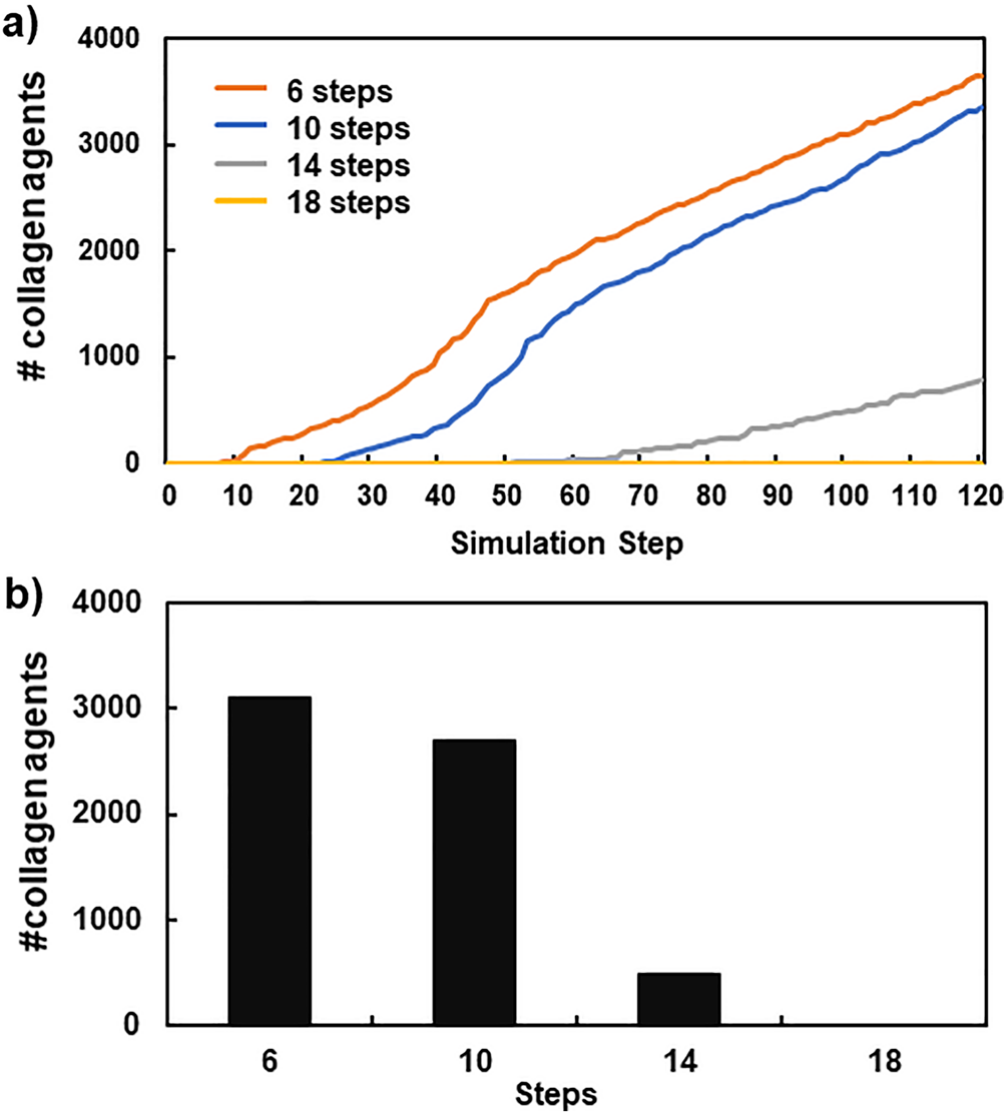
Relationship between the interval of toxic compound injection and the number of produced collagen agents. X and Y-axes indicate the time (step) and collagen amount, respectively (**a**). X and Y-axes indicate the interval and the number of collagen agents, respectively (**b**).

Figure 4 shows the amounts of accumulated collagens under various intervals of toxic compound injection. The tested injection intervals were 6, 10, 14, and 18 steps, and the effects on collagen formation were evaluated. Collagen accumulations became slower and decreased as the interval became longer (Fig. 4a). Furthermore, the simulation at the 18-step interval showed no collagen accumulation (Fig. 4a,b), suggesting that dead cells and HMGB1 were cleared and degraded between long intervals and did not reach the thresholds to activate Kupffer cells.

### Cell death caused by inflammation-initiated liver fibrosis

**Figure 5:**
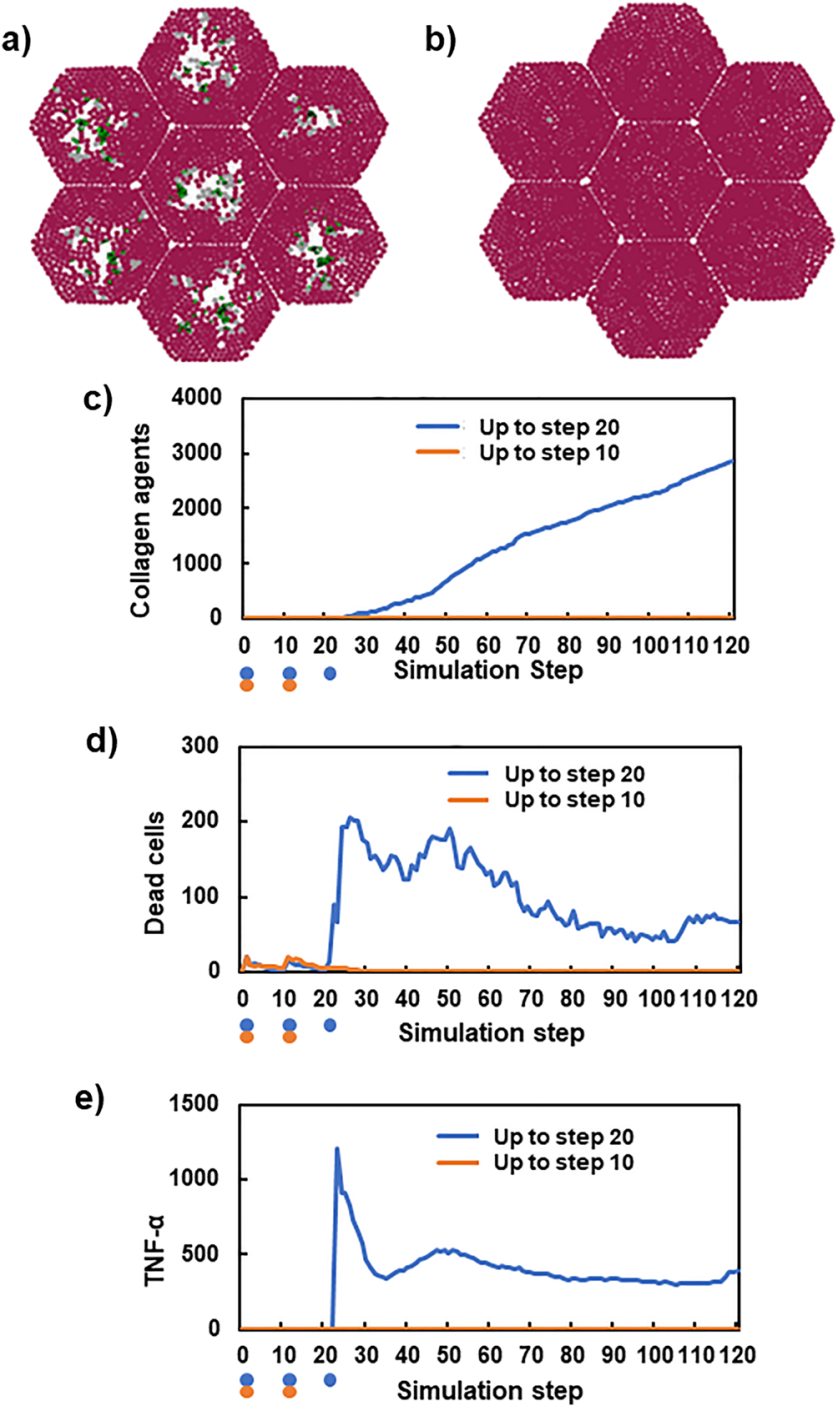
Comparison of the processes of fibrosis between two CCl_4_ injection conditions. Panels (**a**) and (**b**) show simulated liver lobules after 100 steps. CCl_4_ was injected thrice (**a**) and twice (**b**). Hepatocytes (brown), dead cells (grey), and collagens secreted from myofibroblasts and portal fibroblasts (blue and green). The initial conditions before simulation are depicted in Fig. S2. Time courses of the number of collagen agents (**c**), dead cells (**d**), and TNF-α (**e**). Orange and blue circles indicate the timing of CCl_4_ injections.

Figure 5 compares the progression of liver fibrosis between simulations with 2 injections and that with 3 injections of toxic compounds with the same 10-step interval. Initially, there were no dead cells and collagen (Fig. S2). Figure 5a,b show the simulated tissue sections of lobules at 100 steps. After 3 injections of toxic compounds, fibrosis still progressed even after the final injection (Fig. 5a). The time-dependent accumulation of collagens shown in Fig. 5c was consistent with the simulated tissue sections in Fig. 5a,b. Figure 5d shows the time-course of the number of dead cells. When toxic compounds were injected up to step 10 (2 injections), dead cells were not observed after approximately step 20. In contrast, when toxic compounds were injected thrice, the number of dead cells increased rapidly at step 20. The results may represent in vivo observations: the liver could repair the damage before collagens started to accumulate if the damage was limited; however, once the damage exceeded a certain threshold, it appeared to require collagens to maintain the structure and help further repair, which could induce excessive production of collagens and progression of liver fibrosis. Notably, the sharp increase in the number of dead cells was synchronised with the rapid increase of TNF-α concentration (Fig. 5e). This result suggested that the dead hepatocytes caused by inflammation initiated liver fibrosis.

### The ratio of residential liver cells affected progression of liver fibrosis

**Figure 6:**
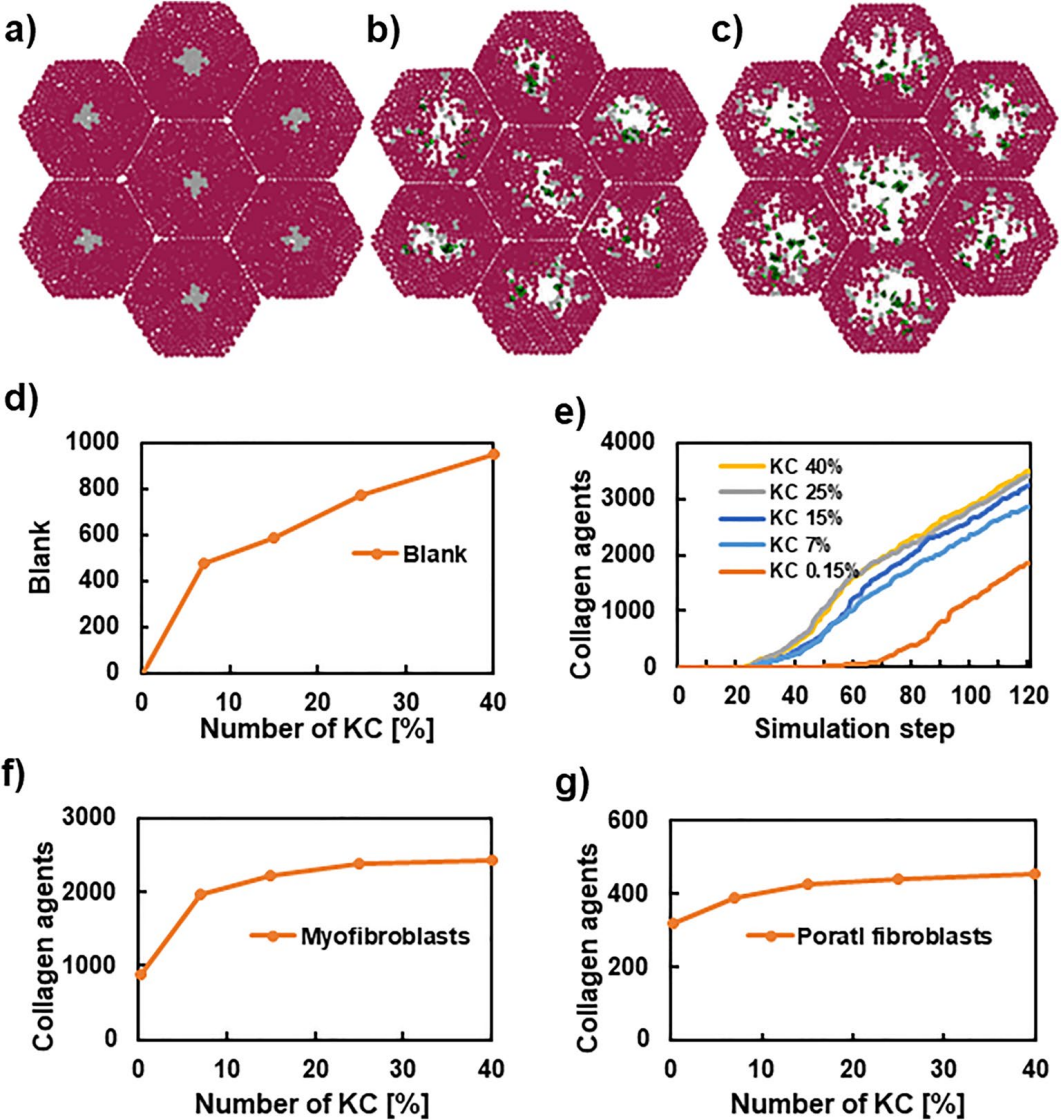
The comparison of simulated results with different initial KC cells. The 2-dimensional tissue sections at step 40 (**a**–**c**). Hepatocytes (brown), dead cells (grey), and collagens secreted from myofibroblasts and portal fibroblasts (blue and green). The initial conditions before simulation are depicted in Fig. S2. Panel (**d**) shows the relationship between the initial number of KC and blank areas. Panel (**e**) shows the time-course of collagen agents with various initial KC ratios. Panels (**f**) and (**g**) show the relationship between initial KC ratios and collagen agents produced from myofibroblasts and portal fibroblasts, respectively.

**Figure 7:**
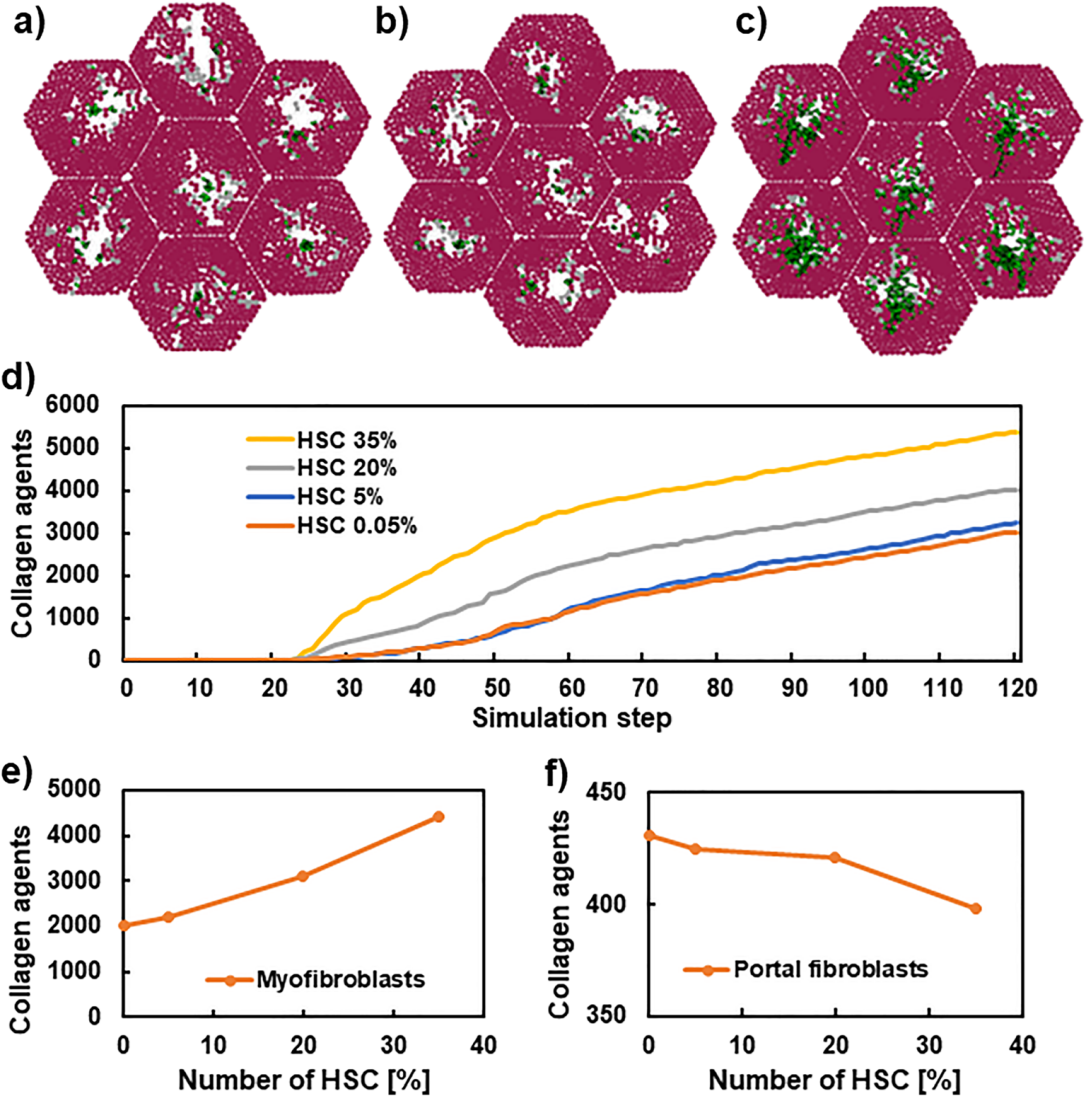
Comparison of simulated results with different initial HC cells. The 2-dimensional tissue sections at 40 steps (**a**–**c**). Hepatocytes (brown), dead cells (grey), and collagens secreted from myofibroblasts and portal fibroblasts (blue and green). The initial conditions before simulation are depicted in Fig. S2. Panel (**d**) shows the time-course of collagen agents with various initial HSC ratios. Panels (**e**) and (**f**) show the relationship between initial KC ratios and collagen agents produced from myofibroblasts and portal fibroblasts, respectively.

Figures 6 and 7 show the effects of the number of residential liver cells on fibrosis progression. The number of accumulated collagens was compared with various initial numbers of KCs and HSCs.

Figure 6a–c shows simulated tissue sections at step 40 with various initial numbers of KCs. Under a 0.15% initial KC ratio of all cells, dead cells remained, and collagens were not produced (Fig. 6a). With a 15% initial KC ratio, dead cells were digested, and collagens were observed (Fig. 6b). In contrast, with a 40% initial KC ratio, although dead cells were cleared and collagens were produced in a similar manner as observed with a 15% initial KC ratio, large areas remained as blank spaces (Fig. 6c).

The relationship between the initial KC ratio to all cells and the blank areas indicated a rapid increase of blank areas between 0.15% and 7% of the initial KC ratio and a moderate increase when KC ratio ≥ 7% (Fig. 6d). These data showed that the lower initial KC ratio resulted in insufficient clearance of deal cells. Alternatively, a higher initial KC ratio resulted in excessive phagocytosis, which led to large blank areas. Figure 6e shows the time-course of collagen agents with various initial KC ratios. When initial KC ratio was 0.15%, collagen production started later, while the other ratios showed similar time courses.

Figure 6f,g shows the amounts of collagens produced by myofibroblasts and portal fibroblasts, respectively, at step 100 with various initial KC ratios. The number of collagens, regardless of the origin, was almost constant for initial KC ratios over 15%. As shown in Fig. 6a–d, high initial KC ratios (≥ 15%) resulted in sufficient clearance of dead cells by phagocytosis, whereas a large blank space with insufficient collagen would not maintain the structure of lobules as observed in Fig. 6c. Collectively, these results indicated that an initial KC ratio of 15% was ideal. Notably, this initial KC ratio was identical to the commonly observed ratio of residential cells in a lobule (hepatocyte: 60%, KC: 15%, HSC: 5%) in the liver.

Figure 7a–c shows simulated tissue sections at step 40 under varying initial HSC population sizes. Larger blank spaces were observed when initial HSC number was low. However, when initial HSC percentage was 35%, production of numerous collagens by myofibroblasts was observed in the centre, even at the early stage of fibrosis. Figure 7d shows the time-course of collagen amounts. A larger initial HSC ratio to all cells resulted in higher collagen production, which was consistent with the results in Fig. 7a–c.

Figure 7e,f shows the accumulated collagen amounts at step 100 under various initial HSC ratios. Higher initial HSC ratios induced higher number of myofibroblast-derived collagens and the low amount of portal fibroblast-derived collagens. The increased number of collagens would contribute to maintaining the structure of the lobules. However, excessive collagen accumulation leads to a decrease in liver function. Therefore, there may be an ideal initial HSC ratio based on the trade-off relationship to determine the appropriate number of collagens.

## Discussion

This model focused on liver fibrosis caused by toxic compounds which undergo metabolic activation. This type of liver fibrosis begins with hepatic injury in central areas, as toxic compounds induce greater metabolic stress in central areas^39^. Collagens are also first produced in the centre area and subsequently spread out to portal areas. Eventually, collagens are bridged especially between centre-centre areas, leading to liver cirrhosis.

Dutta’s model successfully reproduced collagen production in portal areas. However, there was a discrepancy between their simulated collagen production and that experimentally observed in the central vein area. This discrepancy was caused by the following implementations: (1) Myofibroblasts moved to portal areas and produced collagen there. (2) Portal fibroblasts did not produce collagens or contribute to liver fibrosis. Therefore, the previous model would not accurately reproduce collagen production in the central areas of the liver.

Therefore, we modified the model to reproduce fibrosis dynamics more accurately. To eliminate discrepancies, myofibroblasts and portal fibroblasts were separately defined as resources to produce collagens in centre and portal areas, respectively.

Consequently, the simulated and experimentally observed 2D-tissue sections upon toxic compound exposure exhibited highly similar dynamics of collagen formulation. Furthermore, sensitivity analyses revealed the following three features: (1) Liver fibrosis did not progress when the same dosage of toxic compounds was administered in longer intervals because factors inducing fibrosis, such as cytokines, were degraded during intervals and did not accumulate. (2) There was a threshold of the number of dead cells required to initiate liver fibrosis, and it was more susceptible to the concentration of TNF-α than toxic compounds. (3) The balance of the initial cell numbers of KC and HSC would determine collagen production. In silico models have the advantage of allowing for determination of quantitative relationships among multiple components in the liver, although validation of models is mandatory. The complementary use of such models with in vivo and in vitro experiments would contribute to elucidation of complicated biological mechanisms underlying liver damage.

There are limitations to our study. First, the current model could not reproduce the differences of fibrosis caused by various aetiologies as reported by in vivo experiments and clinical observations^40^. To overcome this limitation, cholangiocytes and bile ducts would be an important feature to implement. Besides, a variety of DAMPs depending on different types of cell death may need consideration. Second, the regression process of liver fibrosis was not considered in the current model. The liver has a healing function that induces decomposition of collagens and HSC apoptosis^2^; however, our model did not implement these healing mechanisms. Moreover, some parts of the model may need to be detailed according to in vivo evidence. For example, TNF-α is reported to contribute to the survival of activated HSCs, and potentiate TGF-β signalling; however, it is still unclear if TNF-α can directly transform HSCs to myofibroblasts, as was simplified in this model^41,42^.

Implementing the above-mentioned features would allow for more accurate reproduction of the fibrosis process caused by various pathogeneses. More multifaceted validation also ensures the validity of the mathematical model, contributing to the understanding of the complex mechanisms underlying fibrosis and developing new diagnostic and therapeutic methods.

In summary, we developed a mathematical model that recapitulated liver fibrosis, including the inflammation process in the central vein area. First, toxic compound injection led to collagen production via myofibroblasts in the central vein area. Second, collagens were produced by portal fibroblasts in the portal area. Third, collagens were bridged among central vein areas. Finally, collagens were produced in various areas in liver lobules. These collagen production dynamics were also observed in 2D-tissue section images collected after in vivo experiments. Such simulations using the mathematical model combined with in vivo and in vitro experiments would contribute to understanding the complex pathogenesis of fibrosis.

## Supplementary Information

Supplementary Figures.

## Data Availability

The datasets used and/or analysed during the current study available from the corresponding author on reasonable request.
