# Supplementary material for: Computational simulation of liver fibrosis dynamics
Source: Sci Rep. 2022 Aug 18;12:14112. doi: 10.1038/s41598-022-18123-w (PMC9388486; doi:10.1038/s41598-022-18123-w)
Supplement: Supplementary file 1 — Supplementary Figures. [file 41598_2022_18123_MOESM1_ESM.pdf]

Supplementary Information

Computational simulation of liver fibrosis dynamics

Misa Yoshizawa[1], Masahiro Sugimoto[23], Minoru Tanaka[45], Yusuyuki Sakai[1], Masaki Nishikawa[1]

[1]Department of Chemical System Engineering, University of Tokyo, Tokyo, Japan.
[2]Institute of Medical Science, Tokyo Medical University, Japan
[3]Institute for Advanced Biosciences, Keio University, Yamagata, Japan.
[4]Department of Regenerative Medicine, Research Institute, National Center for Global Health and Medicine, Tokyo, Japan.
[5]Laboratory of Stem Cell Regulation, Institute for Quantitative Biosciences, University of Tokyo, Tokyo, Japan.

*__Corresponding author:__ Masahiro Sugimoto, PhD.
Institute of Medical Science, Tokyo Medical University, 6-1-1, Shinjuku, Tokyo 160-0022, Japan,
Tel: +81-235-29-0528, Fax: +81-235-29-0574
Email: mshrsgmt@tokyo-med.ac.jp, mshrsgmt@gmail.com

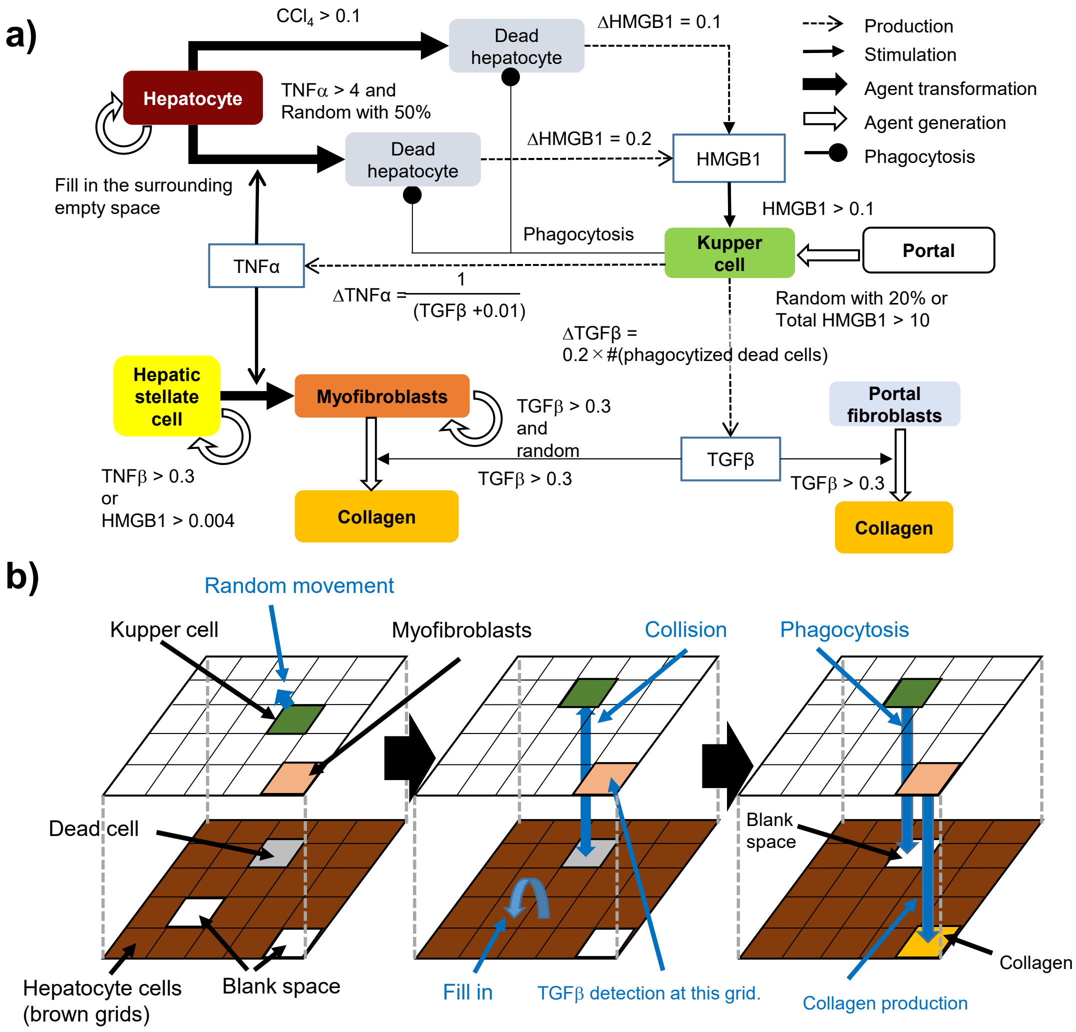

Figure S1 Scheme of the simulation. **a)** Relationship among each agent and molecule in each time step. Agents (squares with rounded corners) include hepatocyte, dead hepatocyte, Kupper cell, myofibroblasts, portal fibroblast, and collagens. The molecules (squares) include HMGB1, TNFα, and TGFβ. The relationships include production, stimulation, agent transformation, agent generation, and phagocytosis. For each production, conditions or incremental values are described. All numerical values have no unit and are used in the context of relative relationships. The process must be initialized before this step. The myofibroblasts, Kupffer cells, and hepatic stellate cells are randomly located on the lobule, and portal fibroblasts are located at portal sites (**Figure S2**). **b)** Example of events in the simulation through three steps. The bottom layer includes value-including cells, such as hepatocytes, dead cells, and collagen. The diffusion of HMGB1 takes in the bottom layer. The upper layer is a virtual space representing the grid address identical to the bottom layer. The diffusion of secreted components such as TNF-α, and TGF-β takes place in this layer. The locations of Kupffer cells, myofibroblasts, and hepatic stellate cells are managed in this layer. Kupffer cells can move randomly to the neighbour grids (chemotaxis). A collision occurs between Kupffer cell and dead cells when they are located in the same address, and Kupffer cell phagocytoses the dead cell, i.e., the dead cell is replaced with a space.

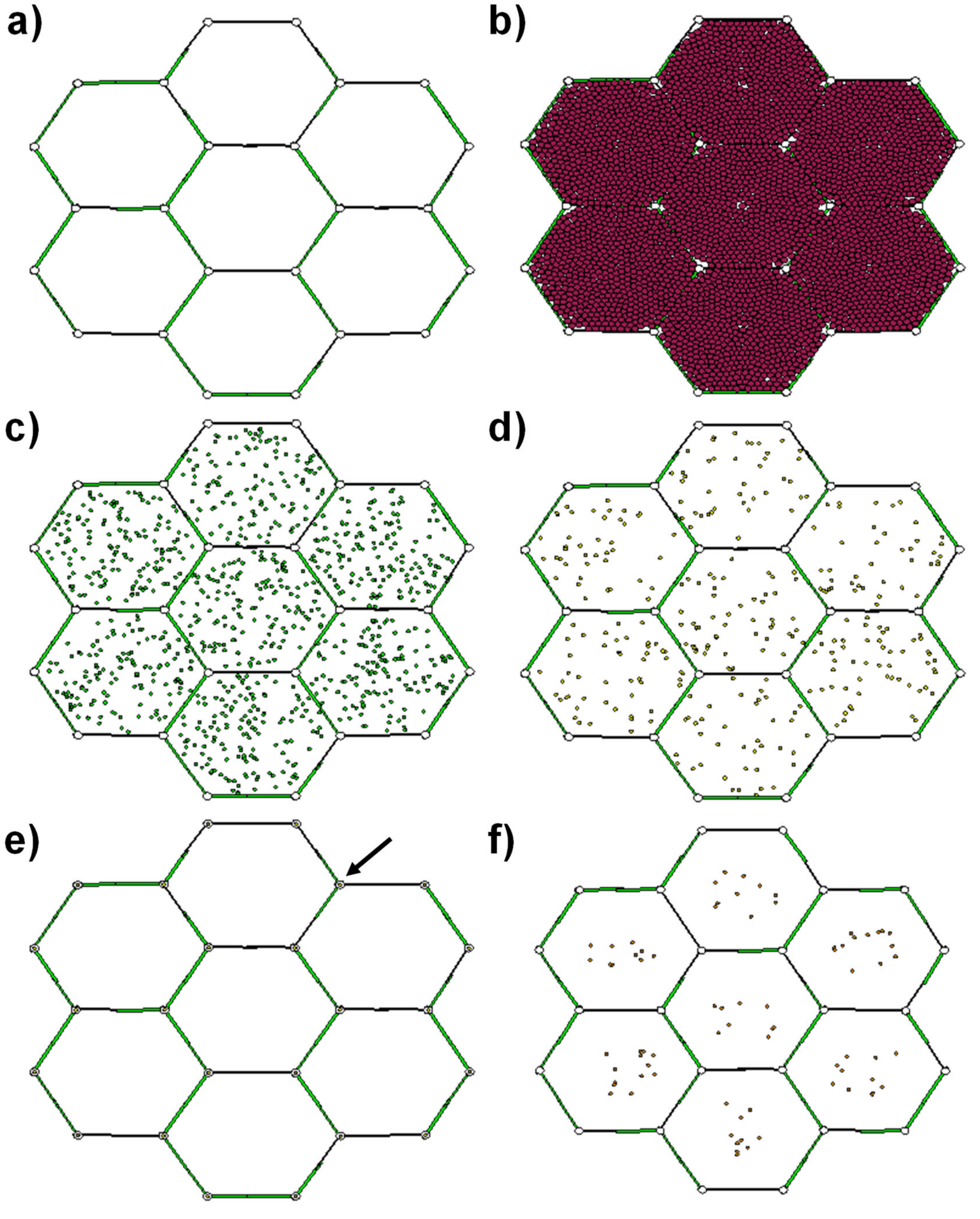

Figure S2 Initial images of 2-dimensional tissue sections of the simulation model. **a)** Boundary lines of liver lobules (green lines) and portal veins (open circles). **b)** Hepatocytes (brown circles). **c)** KC (green circles). **d)** HSC (yellow circles). **e)** Portal fibroblasts (circles at the portal veins). One is indicated by an arrow. **f)** Myofibroblasts (representative result at 25 steps)

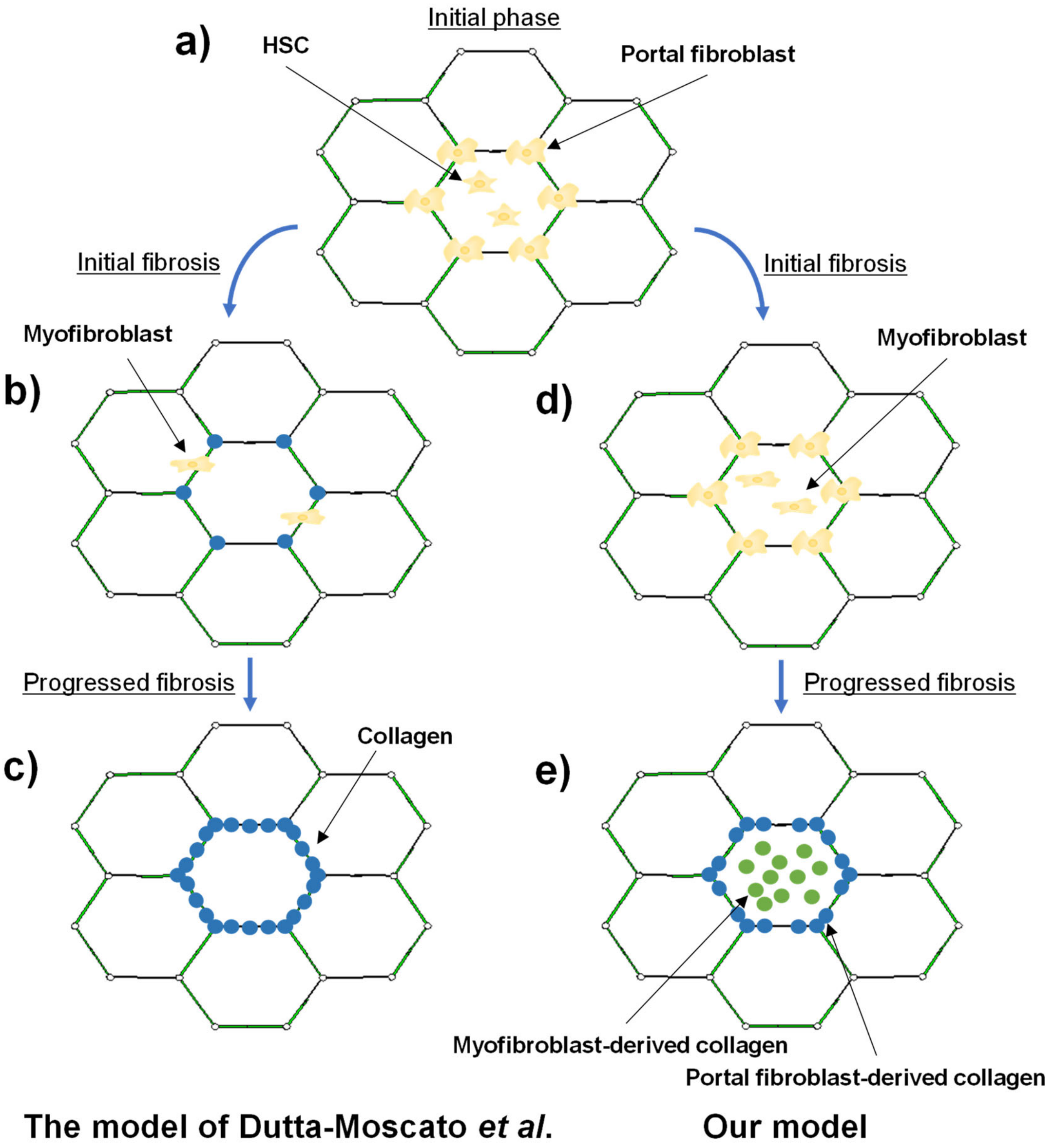

Figure S3 Differences between the mathematical model of Dutta-Moscato *et al* and the one in this study. **a)** Initial status of both models. HSCs are randomly distributed in lobules, and portal fibroblasts are located at the portal veins. **b)** The initial phase of fibrosis of the model by Dutta-Moscato *et al*. Myofibroblasts randomly moved and reached the boundary lines of lobules. **c)** A progressed phase of fibrosis in the Dutta-Moscato *et al*. model. The collagens were produced only at the boundary lines, including portal vein areas. **d)** An initial fibrosis phase in our model. Myofibroblast cells did not move. **e)** A progressed phase of fibrosis of the model in this study. Myofibroblast-derived collagens and portal fibroblast-derived collagens contribute to fibrosis at central vein areas and the portal vein areas, respectively.

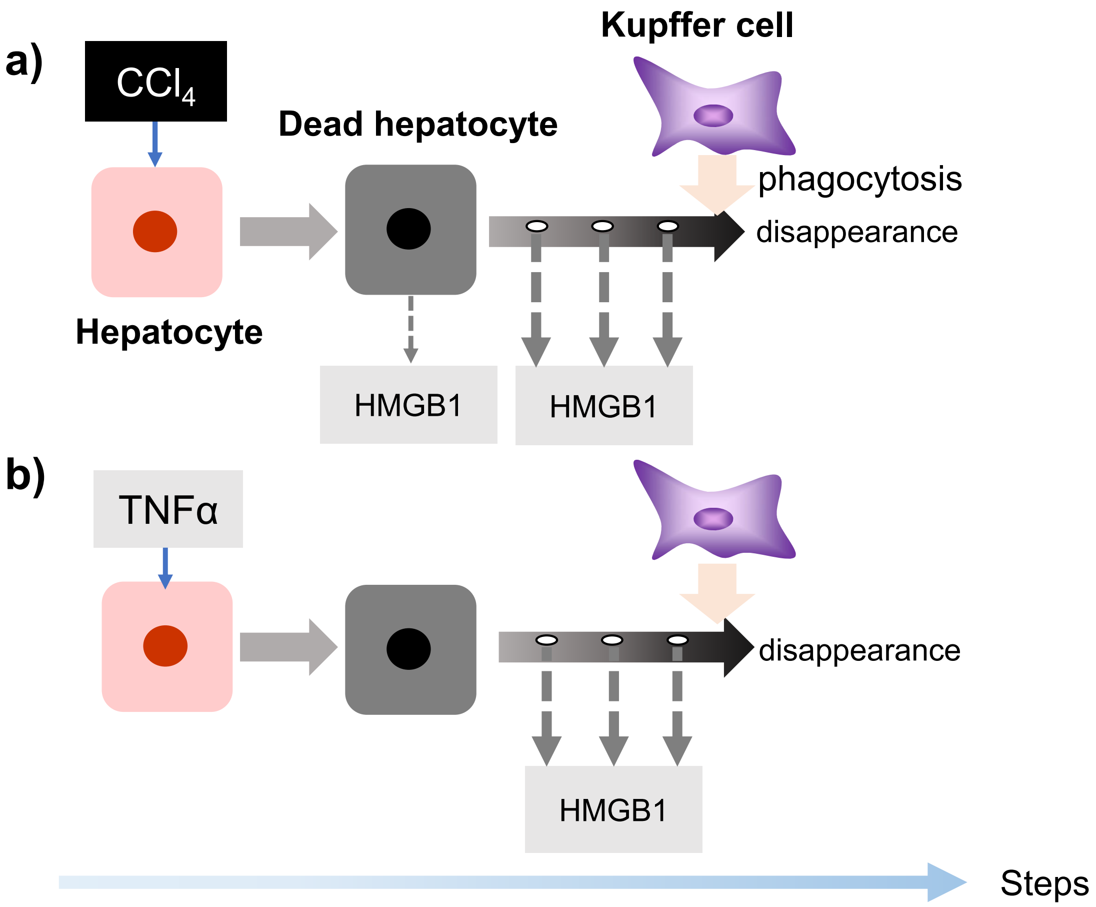

Figure S4 The difference in HMGB1 production from dead hepatocytes between necrosis and apoptosis, considered in this model **a)** When hepatocytes die after CCl_4 exposure (necrosis), they secrete HMGB1. Subsequently, the dead cells also continuously secrete HMGB1 until they disappear via phagocytosis by Kupffer cells. The amount of HMGB1 secreted upon death is half of that secreted afterwards. **b)** During TNF-α-induced hepatocyte death (apoptosis), the cells do not secrete HMGB1. Thereafter, the dead cells secrete HMGB1 repetitively until phagocytosed by Kupffer cells.

Abbreviations.
(in the order of appearance)

agent-based modelling (ABM)
damage-associated molecular patterns (DAMPs)
high mobility group box 1 (HMGB1)
adenosine triphosphate (ATP)
extracellular cold-inducible RNA-binding protein (eCIRP)
heat shock proteins (HSPs)
extracellular RNAs (exRNAs)
cell-free DNA (cfDNA)
transforming growth factor-β (TGF-β)
platelet-derived growth factor (PDGF)
extracellular matrix (ECM)
hepatic stellate cells (HSCs)
tetrachloromethane (CCl_4)
Kupffer cells (KCs)
tumor necrosis factor-α (TNF-α)
